# Prevalence and Predictors of Early Cardiovascular Events after Kidney Transplantation: Evaluation of Pre-Transplant Cardiovascular Work-Up

**DOI:** 10.1371/journal.pone.0131237

**Published:** 2015-06-24

**Authors:** Marianne Delville, Laurent Sabbah, Delphine Girard, Caroline Elie, Sandra Manceau, Marie Piketty, Frank Martinez, Arnaud Méjean, Christophe Legendre, Rebecca Sberro-Soussan

**Affiliations:** 1 Department of Nephrology and Transplantation, Hôpital Necker Assistance Publique-Hôpitaux de Paris, Université Paris Descartes Sorbonne Paris Cité, Paris, France; 2 Department of Cardiology, Hôpital Necker, APHP, Paris, France; 3 Department of Biostatistics, Hôpital Necker, APHP, Paris, France; 4 Université Paris Descartes, Sorbonne Paris Cité, RTRS « Centaure », Labex « Transplantex », Paris, France; 5 Department of Clinical Research, Hôpital Necker, APHP, Paris, France; 6 Department of Functional Explorations, Hôpital Necker, APHP, Paris, France; Medical University of Graz, AUSTRIA

## Abstract

**Introduction:**

Cardiovascular disease is the leading cause of mortality after renal transplantation. The purpose of this study was to analyze cardiovascular risk factors at transplantation, occurrence of cardiovascular events in the first year after transplantation and evaluate pre-transplant work-up.

**Material and Method:**

In total, 244 renal transplant recipients older than 50 years were included. The results of pre-transplant work-up, including clinical evaluation, electrocardiogram, echocardiography, myocardial perfusion testing and coronary angiography were analyzed.

**Results:**

Patients had multiple risk factors at inclusion on renal transplantation waiting list as high blood pressure (94.7%), dyslipidemia (81.1%), smoking (45.3%), diabetes (23.6%), past history of cardiovascular disease (21.3%) and obesity (12.7%). Following transplantation, 15.5% (n = 38) of patients experienced a cardiovascular event, including 2.8% (n = 7) acute coronary syndrome, 5.8% (n = 14) isolated increase in troponin level and 5.3% (n = 13) new onset atrial fibrillation. The pre-transplant parameters associated with a cardiovascular event were a past medical history of cardiovascular disease (HR = 2.06 [1.06–4.03], p = 0.03), echocardiographic left ventricular hypertrophy (HR = 2.04 [1.04–3.98], p = 0.037) and abnormal myocardial perfusion testing (HR = 2.25 [1.09 –5.96], p = 0.03). Pre-transplantation evaluation allowed the diagnosis of unknown coronary artery lesions in 8.9% of patients.

## Introduction

Cardiovascular disease remains the leading cause of mortality after renal transplantation. Overall, 47% of deaths without kidney failure in the first month post-transplantation are related to cardiovascular disease[1,2,3,4]. The cumulative incidence of acute coronary syndrome (ACS) is between 7 and 11% at 3 years after transplantation[5,6,7]. This initial increase in cardiovascular event occurrence is related to surgical procedure and peri-operative period of time[8,9,10].

Renal transplant recipients present not only with various traditional risk factors, such as diabetes, high blood pressure, smoking and history of coronary artery disease[11], but also with more specific risk factors related to end-stage renal disease (ESRD), such as endothelial dysfunction, calcemia and phosphoremia imbalance, anemia and variations in fluid overload following hemodialysis[7,12,13,14,15].

Prior to transplantation, a cardiovascular evaluation is highly recommended by KDIGO guidelines, including clinical evaluation, electrocardiogram (ECG) and cardiac echocardiography[9,16]. Invasive testing is recommended for patients presenting clinical symptoms of coronary ischemia. However guidelines show discrepancies concerning non-invasive testing and are mostly not written specifically for ESRD patients. Based on an AHA 2012 statement, non-invasive testing should be considered for patients showing more than three risk factors[9,17]. Of all available non-invasive testing, myocardial perfusion imaging is well validated for ESRD patients[18,19,20,21]. The negative predictive value of myocardial perfusion imaging (MPI) of renal transplant recipients ranges from 0.61 to 0.98[18,22,23].

Considering aging of ESRD population, high prevalence of cardiovascular disease, large size of kidney transplant waiting lists and increasing waiting time, there is an urgent need for an efficient, cost-effective screening strategy. At our center, we perform non-invasive screening, mostly with myocardial perfusion imaging, of all patients older than 50 years at inclusion. The purpose of this study was to evaluate prevalence of cardiovascular risk factors, prevalence of cardiovascular events during the first year post-transplantation and prognostic factors of early cardiovascular events after kidney transplantation including the prognostic value of our pre-transplant cardiac work-up.

## Materials and Methods

### Population

The only inclusion criterion was age over 50 years on the day of listing. Combined kidney-liver transplant recipients were excluded because early follow-up was not performed in our department. We included both living and cadaveric donor recipients and preemptive transplantation. Overall, 244 renal transplant recipients were included between January 1^rst^, 2005, and December 31^rst^, 2009.

### Ethics Statement

Patients’ data were extracted from the DIVAT (Données Informatisées et Validées en Transplantation) clinical prospective cohort database. All patients received information and gave written consent. Codes were used to ensure anonymity. The quality of DIVAT data bank is validated by an annual cross-center audit. Approval was obtained at the French Commission Nationale Informatique et Liberté (www.divat.fr, n° CNIL 891735, August 2004).

### Evaluation

We retrospectively collected data on pre-transplantation clinical and biological parameters, treatments and clinical and biological follow-up during the first year after transplantation.

Recipients’ demographic characeristics were collected, including age, gender, nephropathy, time spent on a waiting list and on dialysis.

All traditional risk factors were collected as follows: age at transplantation, body mass index (BMI), quantification of past or active smoking history and past medical history of coronary artery disease.

Diabetes definition was as a past medical history of diabetes or a glycated hemoglobin (HbA1c) level above 6.5% at admission. Treatment categories were as follows: diet, oral anti-diabetic treatment and insulin. The definition of dyslipidemia was a past medical history of dyslipidemia or an LDL-cholesterol level above 2.6 mmol/l at inclusion. Treatments were as follows: diet, statins and fibrates. Hypertension was defined as a past medical history of high blood pressure, a blood pressure level of 140/90 mmHg or higher at admission. Treatments were as follows: beta-blockers, calcium inhibitors, angiotensin 2 receptor antagonists and conversion enzyme inhibitors, diuretics and other. Exposition to biochemical anomalies included evaluation of calcium-phosphorus product, PTH and 25-OH-D3 at transplant. Baseline troponin level at transplant was recorded. Data on prescriptions for an anti-platelet agent for primary or secondary prevention were collected.

Cardiovascular evaluation before transplantation was recorded. Electrocardiography was deemed normal or showing left ventricular hypertrophy (LVH), repolarization conduction or rhythm trouble. Electrocardiographic LVH was diagnosed following Sokolow-Lyon criteria when the voltage amplitude sum of either SV1 + RV5 or SV1 + RV6 at baseline was equal to/or above 3.5 mV. Cardiac echography results were classified as normal, showing LVH if the left ventricular mass was above 134 g/m^2^ in men or above 125 g/m^2^ in women or kinetic trouble. Mean Left atrium at pre-transplant echocardiography was measured and considered abnormal if above 40 mm. Myocardial perfusion imaging was classified as normal, pathologic if two or more territories showed hypoperfusion or non-contributive if targeted heart rate of was not reached or if a drug interaction was shown. Several patients underwent coronary angiography. Data on indications, coronary artery lesions and treatment were collected.

During the early post-transplantation period (15 days following transplantation), the following features were collected: biological parameters (troponin, creatinine nadir, C-reactive protein and hemoglobin levels), immunosuppressive regimen, delay to reintroduction of beta-blockers and aspirin after surgery.

Data on cardiovascular event occurrence were exhaustively collected up to 1 year of follow-up. The events were defined as follows: 1: acute coronary syndrome (ACS) associated with ST-segment elevation (>2 mm) and increased troponin level, 2: ACS without ST-segment modification and troponin elevation, 3: new onset atrial fibrillation or ventricular fibrillation, 4: death from cardiovascular disease. Blood pressure, cardiac frequency, weight increase, serum creatinine levels, potassium serum levels, hemoglobin levels, troponin levels and ECG and echocardiography results were recorded at the time of any cardiovascular event.

### Statistical analysis

Results are expressed as numbers and percentages for categorical variables and as a mean (± standard deviation) or median [range] for continuous variables.

Survival curves were computed by the Kaplan-Meier method and compared by the log-rank test to select prognostic factors of cardiovascular events occurring during the year following the kidney transplantation. Patients who died during the follow-up for extra-cardiac causes were censured. Cox regression models were used for multivariate analyses. P value <0.10 was used for the variable entry criteria. Then a backward selection procedure was applied. Only, the factors associated with the considered outcome with P value <0.05 were kept in the final model. A forward selection procedure was used to control the robustness of the final model.

Statistical analysis was performed using the R package Version 2.10.

## Results

### Patient characteristics

A total number of 244 patients were included. Table 1 shows patients demographics characteristics. Clinical evaluation revealed a high prevalence of traditional risk factors, with 230 (94.7%) patients with high blood pressure, 172 over 214 patients (81.1%) with dyslipidemia, 110 (45.3%) with a smoking history and 49 over 208 patients (23.6%) with diabetes. A BMI of 30 and above was reached by 26 patients out of 233 (12.7%). Overall, 52 (21.3%) patients had a past medical history of coronary artery disease. HbA1c levels were not in the therapeutic target range in 30 (14.7%) diabetic patients. Only 115 (66.8%) over 137 dyslipidemic patients received a medical treatment of dyslipidemia at inclusion. LDL-cholesterol levels were above 2.6 mmol/l for 75 (30.7%) patients. In addition to ESRD and age above 50 years, 151 (61.9%) patients had more than four cardiovascular risk factors before transplantation. Overall, 63 (25.8%) patients were treated with an anti-platelet agent. Biochemical evaluation at transplant showed mean calcemia level of 2.3 ± 0.2 mmol/l, phosphatemia 1.5 ± 0.5 mmol/l, calcium-phosphorus product 3.4 ± 1.1 mmol^2^/l^2^, PTH level 241.5 ng/l [7–1770] and 25-hydroxyvitamin D 17.0 ng/ml [5–63].

**Table 1:** Patients’ characteristics at inclusion.

Pre-transplantation screening	Population (n = 244)
Gender, male, % (n)	55.3 (135)
Age at inscription time, years, median [range]	58.0 [50–81]
Time on waiting list, years, median [range]	2 [0.02–10]
Time on dialysis, years, median [range]	3.6 [0.0–40.5]
History of cardiovascular disease, % (n)	21.3 (52)
High blood pressure, % (n)	94.7 (230)
Dyslipidemia, % (n)	81.1 (172)
BMI at inscription, kg/m2, median [range]	24.6 [20.3–28.9]
Smoking history, %(n)	45.3 (110)
Calcemia, mean mmol/l (± sd)	2.3 ± 0.2
Phosphoremia, mean mmol/l (± sd)	1.5 ± 0.5
Phosphocalcic product, mean mmol2/l2 (± sd)	3.4 ± 1.1
PTH, median, ng/l [range]	241.5 [7–1770]
25-OH-D3, median, ng/ml [range]	17.0 [5–63]

With regard to immunosuppressive regimen: 211 (86.5%) patients received an combination of anti-CD25 (Basiliximab) 20mg at day 0 and 4, steroids 500mg at day 0, 125mg at day 1 then rapidly tapered to 10mg per day, calcineurin inhibitors (cyclosporine or tacrolimus) and antimetabolites. Highly immunized patients (13.5%) received induction with Thymoglobulin (1.5mg/kg/day for five days) and intravenous immunoglobulin (four courses of 2g/kg).

### Pre-transplant cardiovascular screening

The results of pre-transplant evaluation are shown in Table 2. ECG was normal in 198 (81.5%) patients. We reported repolarization abnormalities in 19 (7.8%) patients, conduction anomalies in 14 (5.8%), arrhythmia in 9 (3.7%) and LVH in 3 (1.3%). Echocardiography (n = 238) determined a mean left ventricular ejection fraction (LVEF) of 62.3% ± 8.3% and identified LVH in 120 (50.4%) patients. Echocardiography is more sensitive compared to ECGs to detect LVH.

**Table 2:** Pre-transplant cardiovascular evaluation and results.

Pre-transplantation workup	Population (n = 244)
**Electrocardiogram results**, % (n)	100.0 (244)
Normal	81.5 (198)
Repolarization abnormality	7.8 (19)
Conduction abnormality	5.8 (14)
Arrhythmia	3.7 (9)
Left ventricular hypertrophy (LVH)	1.24 (3)
**Echocardiography**, % (n)	97.5 (238)
Normal	11.5 (28)
Left ventricular hypertrophy	50.4 (120)
**Myocardial perfusion imaging**, % (n)	81.1 (198)
Normal	81.8 (162)
Ischemic lesions	11.5 (28)
**Coronary angiography, % (n)**	24.5 (60)
Normal	35.0 (21)
Significant coronary arteries stenosis	65.0 (39)
Revascularization procedure	35.0 (21)
**Stress electrocardiogram, % (n)**	3.7 (9)
Normal	55.5 (5)
Ischemic lesions	44.4 (4)
**Stress echography, % (n)**	1.5 (4)
Normal	50.0 (2)
Ischemic lesions	50.0 (2)
**CT scan, % (n)**	0.4 (1)
**MRI, % (n)**	0.4 (1)

All renal transplant recipients underwent electrocardiography, echocardiography and non-invasive testing.

Myocardial perfusion imaging (MPI) was performed in 198 (81.1%) renal transplant recipients disclosing ischemic lesions in 30 (15.2%) patients. Coronary angiography was performed in 60 (24.5%) patients. Per procedure revascularization was required in 21 (35.0%) patients. However, when coronary angiography was performed due to abnormal scintigraphy, revascularization indication increased up to 50%. No coronary arteries bypass graft surgery was proposed.

Among 182 asymptomatic patients without a past history of cardiovascular disease, 158 (86.8%) myocardial perfusion imaging (MPI) was performed that was negative in 134 (73.6%). MPI diagnosed unknown coronary artery disease in 14 (8.9%) patients. ACS occurred in 3 of these patients.

Overall, 52 (21.3%) patients had a past medical history of cardiovascular disease. Of these patients, 41 (78.8%) were subjected to MPI. Perfusion anomalies were shown in 24 (58.5%) of these patients, leading to coronary angiography. Per-procedure revascularization of coronary arteries was performed in 10 (41.7%) patients.

### Post-transplantation management

A total number of 109 patients (including 92% of patients receiving a beta-blocker before transplantation) had early beta-blocker re- or introduction. Median time to reintroduction was 2 days after surgery. Median time of anti-platelet therapy was 10 days after surgery in 50 (72.5%) patients previously receiving anti-platelet agent for primary or secondary prevention. Prophylactic treatment with heparin was administered to 43 (62.3%)renal transplant recipients.

Serum creatinine nadir was 137.6 μmol/l ± 82.7. The lowest hemoglobin level in the first 10 days was 8.5 g/dl ± 1.2.

### Cardiovascular events

**Fig 1:**
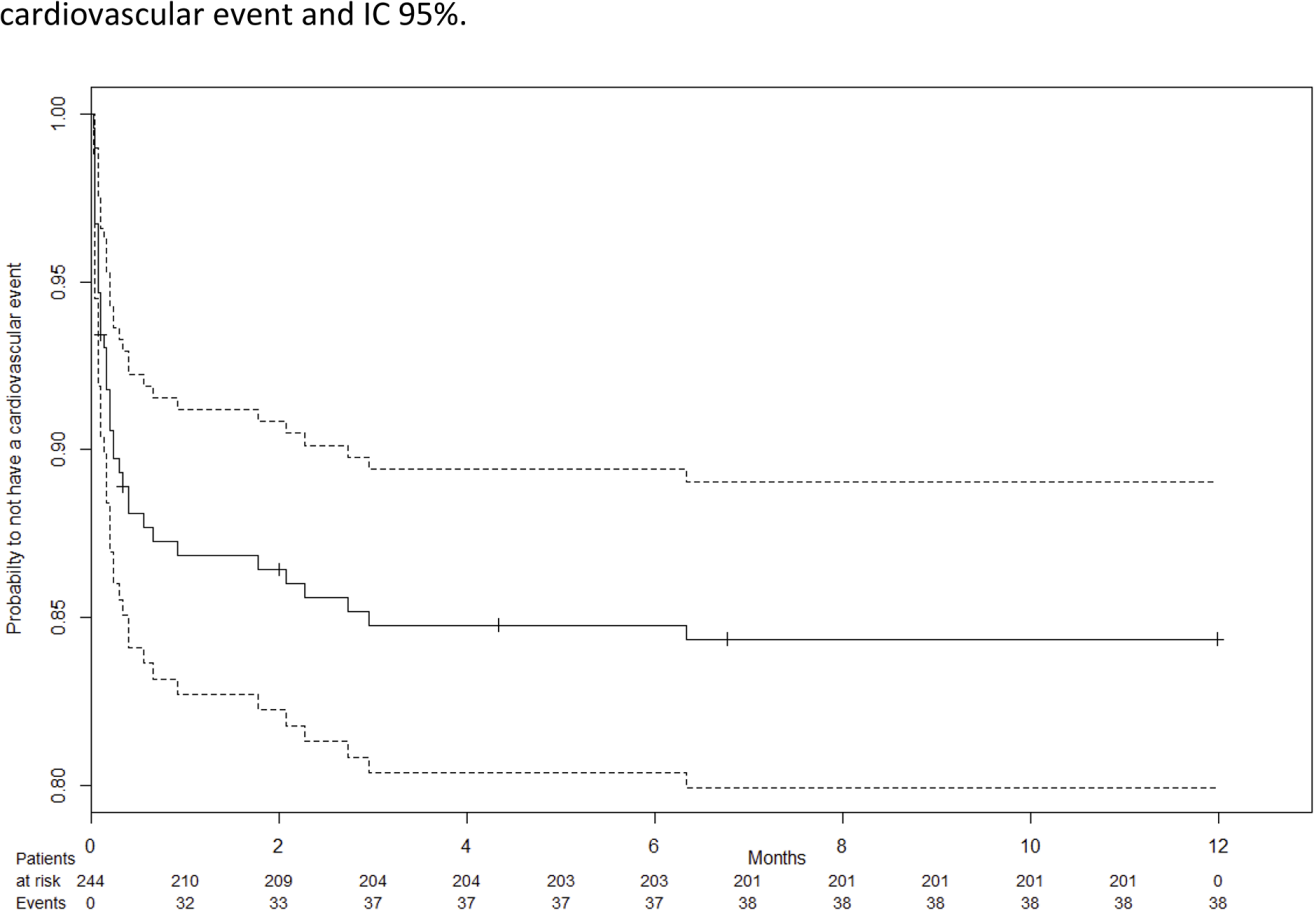
Kaplan-Meier survival curve shows the probability over time to observe a cardiovascular event and IC 95%.

A 12 months, follow-up was available for all patients. Overall, 38 (15.5%) renal transplant recipients had a cardiovascular event during the first year post-transplantation as follows: ACS with ST-segment elevation and increased troponin levels in 7 patients (2.8%), ACS without ST-segment elevation in 14 (5.8%), acute pulmonary edema associated with ACS without ST-segment elevation in 4 (1.6%) and atrial fibrillation in 13 (5.3%). One patient was resuscitated following cardiac arrest related to ventricular fibrillation. Five patients died of non cardiovascular causes (4 sepsis and 1 intracerebral tumor). Kaplan-Meier survival curve (Fig 1) showed the probability over time to observe a cardiovascular event. The probability to have a cardiovascular event was 16% [11%–20%]. The median time of cardiac event occurrence in these 38 patients was 5 days [2–12]. Patients with ACS with ST-segment modification were all men. The median age was 59 years old [51–66]. Mean hemoglobin level in these patients event was 9.8 g/dl ± 1.6. Patients presenting a SCA with ST-segment modification had a trend to more delayed graft function with a serum creatinine at event 613 ± 258 μmol/l vs 433 ± 278 μmol/l and an increased of overload syndrome with an increase of body weight of 3.58 ± 3.23 kg vs 2.93 ± 3.3 kg compared to other event. These results are not significant because of population’s small size. Mean left atrium at pre-transplant echocardiography was 39,5 ± 6.6 mm and was above 46 mm in 6 patients. Among the 6 patients showing aortic valves calcifications at pre-transplant work-up 3 observed atrial fibrillation.

A total number of 22 patients had no instrumental evaluation of cardiac damage. Fifteen of these patients were women. Mean age at listing was 56 ± 6 years. Regarding cardiovascular risk factors: 2 of these patients were obese, 2 overweighted, 4 had diabetes (2 treated by insulin, one by oral anti-diabetic medication, 1 by dietetic restriction), 10 had dyslipidemia, 21 a past history of high blood pressure, none had a past history of cardiovascular disease. LVH was observed in 13 out of them, 2 had relaxation abnormalities. A cardiovascular event was observed in 3 of these patients. One had a troponion level increase with no ST-segment modification, 2 presented with arrhythmia. All of these 22 patients were evaluated and listed for kidney transplantation between 2005.

### Pre-transplant prognosis factors

Univariate analysis of factors associated with early cardiovascular events is presented in Table 3. A past medical history of cardiovascular disease (HR = 2.06 [1.06–4.03], p = 0.03), left ventricular hypertrophy (HR = 2.04 [1.04–3.98], p = 0.04) and abnormal myocardial perfusion imaging (HR = 2.18 [1.00–4.79], p = 0.05) were associated with a higher risk of early cardiovascular event. The absence of indication of coronary angiography after pre-transplantation cardiovascular work up was protective (HR 0.45 [0.21–0.95], p = 0.04). The increase level of phosphocalcic product and PTH were not significant risk factors of CV events. The immunosuppressive regimen had also no impact on these events.

**Table 3:** Analysis of factors associated with early cardiovascular events after kidney transplantation in univariate analysis.

Variable	Hazard Ratio	CI 95%	p-value
**Gender (reference = Male)**	**1.44**	**[0.74–2.78]**	**0.28**
**Age at inscriptiona**	**1.12**	**[0.89–1.42]**	**0.34**
**Age at transplantationa**	**1.14**	**[0.89–1.47]**	**0.31**
**Time on dialysisa**	**1.12**	**[0.91–1.37]**	**0.28**
**High blood pressure**	**1.14**	**[0.59–2.19]**	**0.12**
**Diabetes**	**0.78**	**[0.34–1.79]**	**0.56**
**Glycated Hemoglobin (reference = <7.5%)**	**2.43**	**[0.74–7.96]**	**0.14**
**Obesity**	**0.17**	**[0.023–1.24]**	**0.08**
**Dyslipidemia**			
**Dyslipidemia**	**1.42**	**[0.55–3.66]**	**0.47**
**Total cholesterol>6 mmol/L**	**0.88**	**[0.31–2.48]**	**0.81**
**HDL > = 1 mmol/L**	**1.49**	**[0.62–3.6]**	**0.68**
**LDL >2.6 mmol/L**	**1.12**	**[0.52–2.39]**	**0.77**
**Triglycerides>1.7 mmol/L**	**1.25**	**[0.65–2.4]**	**0.51**
**Vitamine D <30 ng/ml** ^**a**^	**0.52**	**[0.18–1.48]**	**0.22**
**Phosphocalcic product mmol2/l2** ^**b**^	**1.17**	**[0.89–1.53]**	**0.26**
**PTH <350 ng/l** ^**c**^	**0.60**	**[0.29–1.23]**	**0.16**
**Smoking history**	**0.70**	**[0.36–1.35]**	**0.29**
**History of cardiovascular disease**	**2.06**	**[1.06–4.03]**	**0.03**
**Anti-platelet agent treatment**			
**Anti-platelet agent treatment**	**2.21**	**[1.16–4.18]**	**0.02**
**Antiplatelet agent indication (reference = no indication)**			**0.05**
**As primary prevention**	**2.21**	**[0.83–5.90]**	**0.11**
**As secondary prevention**	**2.68**	**[1.31–5.49]**	**0.007**
**Antiplatelet agent indication with no prevention prescription**	**0.88**	**[0.12–6.56]**	**0.91**
**Echocardiography**			
**Abnormal ECG result**	**0.60**	**[0.29–1.23]**	**0.16**
**LVH**	**2.04**	**[1.04–3.98]**	**0.04**
**ETT FEVG (reference = > = 55)**	**0.46**	**[0.19–1.09]**	**0.08**
**ETT normal kinetic**	**0.54**	**[0.23–1.29]**	**0.17**
**Myocardial perfusion imaging (reference = normal)**			**0.18**
**Abnormal**	**2.55**	**[1.09–5.96]**	**0.05**
**Not performed or older than 3 years**	**1.09**	**[0.50–2.39]**	**0.83**
**Thallium delay before transplantation (reference = within the 3 past years)**			**0.87**
**No thallium or older than 3 years**	**0.96**	**[0.44–2.10]**	**0.92**
**Delay less than 1 year**	**1.21**	**[0.55–2.67]**	**0.64**
**Coronary arteriography (reference = within the 3 past years)**			
**No coronary arteriography or older than 3 years**	**0.45**	**[0.21–0.95]**	**0.04**
**Revascularization**	**1.22**	**[0.40–3.74]**	**0.72**
**Troponin at transplantation**	**0.08**	**[4e-5–141]**	**0.51**
**Immunosupressive therapy HIR (Ref. LIR)**	**1.52**	**[0.67–3.46]**	**0.32**
**Post-transplantation follow-up**			
**Beta-blockers reintroduction**	**1.23**	**[0.58–2.62]**	**0.59**
**Antiplatelet agent reintroduction**	**1.85**	**[0.52–6.52]**	**0.34**
**Cumulative cardiovascular risk factors (added to ESRD and age)**			**0.84**
**3**	**0.61**	**[0.21–1.75]**	**0.36**
**4**	**0.75**	**[0.30–1.87]**	**0.54**
**5**	**0.56**	**[0.18–1.70]**	**0.30**
**Above 6**	**0.81**	**[0.26–2.47]**	**0.71**

Immunosuppressive regimen: **LIR = Low immunological risk** = Immunosuppressive therapy, included a combination of anti-CD25 (Basiliximab) 20mg at day 0 and 4, steroids 500mg at day 0, 125mg at day 1 then rapidly tapered to 10mg per day, calcineurin inhibitors (cyclosporine or tacrolimus) and antimetabolites; **HIR = High immunological risk** (immunized patients) = immunosuppressive therapy included induction with thymoglobulin (1.5mg/kg/day for five days), steroids 500mg at day 0, 125mg at day 1 then rapidly tapered to 10mg per day, calcineurin inhibitors (cyclosporine or tacrolimus), antimetabolites, intravenous immunoglobulin (four courses of 2g/kg).

^a^For an increase of 5 units

^b^For an increase of 1 unit

^c^For an increase of 10 units

Multivariate analysis did not allowed to show independent prognostic factors because only LVH shown by echocardiography remained in the model.

## Discussion

Renal transplant recipients represent a population at very high risk of cardiovascular disease. At our center, 62% of patients older than 50 years exhibited more than four traditional risk factors. As previously shown[24], we confirmed that ESRD patients are not evaluated sufficiently thoroughly for coronary disease. Moreover, these patients were undertreated. Indeed, HbA1c levels were in the therapeutic range in only 35% of patients, and dyslipidemia was treated in only 67% of patients. Seventy eight patients had a PTH higher than 350 ng/l. Although 52 (21%) renal transplant recipients had a past medical history of coronary artery disease and 151 (62%) patients had more than four cardiovascular risk factors, only 63 (26%) received anti-platelet agents. Moreover, in 8.9% of patients, coronary artery disease was diagnosed at the time of pre-transplant work-up.

Acute coronary syndrome with ST segment-elevation in the first year after transplantation was observed in 2.8% of our patients aged 50 years and older. The predicted incidence of post-transplantation cardiovascular ACS ranged from 7 to 9%[25,26,27] in different studies, depending on patients’age and diabetic status. ACS occurred in the first 20 days after transplantation highlighting the specific increased of cardiovascular risk related to surgical procedure. Patients presenting with a cardiovascular event had a trend to more delayed graft function and overload syndrome. Considering this association a closer monitoring should be proposed for patients with delayed graft function.

Overall incidence of ACS was low in our study. Prevention of post-transplantation cardiovascular events often depends on perioperative management. Indeed, early beta-blocker reintroduction has been shown to be protective in numerous studies[9,25,26,28]. In our study, beta-blockers were started 2 days after surgery. Anti-platelet agents were reintroduced as soon as possible depending on specific indications for each patient given the bleeding risk of this specific surgery[9,26,29]. Moreover, perioperative management of anemia, fluid intake and blood pressure is essential to prevent perioperative cardiovascular events[26].

This study shows that past history of cardiovascular disease, echocardiography showing LVH and abnormal MPI could be associated with an increase in cardiovascular event occurrence during the first year following transplantation. These parameters are known to be associated with ongoing cardiovascular disease^30,31^. LVH, is the only prognostic factor of cardiovascular events remaining in the multivariate analysis. It is known to be a marker of poor prognosis in ESRD patients[30,31]. Sharma and al. demonstrated that LVH and ischemia on a stress ECG are independent prognosis factors of mortality in patients[32]. One could argue that there is a major discrepancy between ECG and Echocardiography in diagnosis of LVH in our study. ECG had a low sensibility of 6.9% compared to for echocardiography and a good specificity of 98.8%[33].

In our study, MPI diagnosed coronary artery disease in 8.9% of our patients. MPI is a functional evaluation of coronary arteries. Coronary angiography has been considered as the best predictor of cardiovascular events in ESRD patients[27]. However, MPI has been validated among ESRD patients[18,19,20,21]. MPI is a strong predictor of all-cause mortality in patients with ESRD[34]. MPI has a good negative predictive value and its implementation in the pre-transplant work-up is a cost-effective strategy. More recently, MRI associating morphologic and functional evaluation has been proposed for cardiovascular screening[35]. However, description of numerous cases of nephrogenic systemic fibrosis following gadolinium injection in the context of renal insufficiency restrains the MRI indication in ESRD patients[36].

An AHA 2012 statement[9] suggests that non-invasive testing should be proposed to patients with no active cardiac condition who present three or more risk factors, such as diabetes, prior cardiovascular disease, 1 year or more on dialysis, LVH, an age above 60 years old, smoking, hypertension and dyslipidemia. The NKF/KDOQI guidelines[37] recommend that non-invasive testing be performed every 12 months for all patients with diabetes or a past history of cardiovascular disease and every 24 months for non-diabetic patients with two or more traditional risk factors, an LVEF above 40% or peripheral vascular disease. Our results are consistent with these recommendations because we observed that LVH, a past history of cardiovascular disease and abnormal MPI result in a higher risk of cardiovascular events after transplantation.

**Fig 2:**
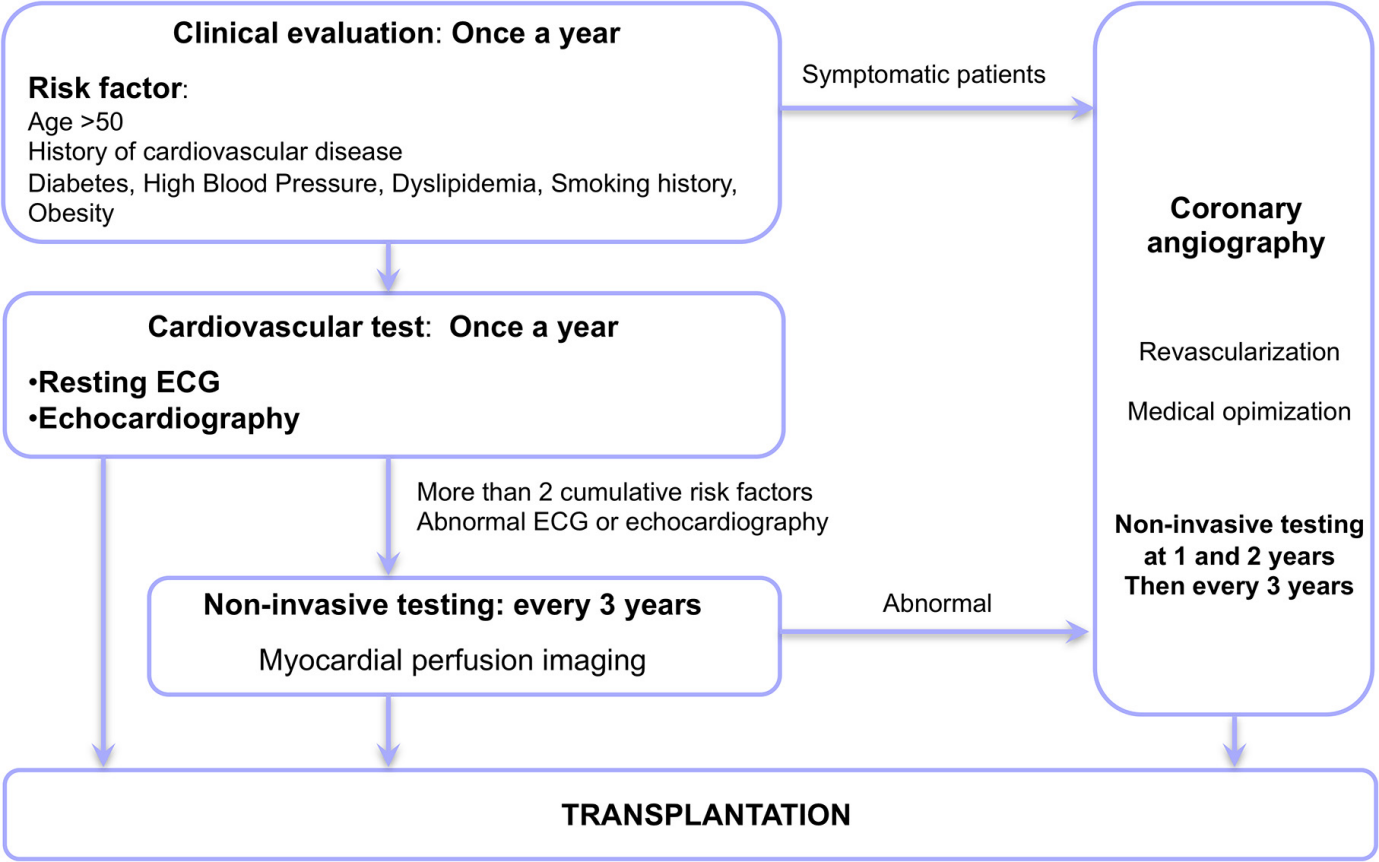
Screening strategy for cardiovascular disease before transplantation.

Based on guidelines and our observations, our screening algorithm is shown in Fig 2. All patients underwent clinical evaluation every 12 months, including an ECG and resting echocardiography[9,31,32,37,38]. Hoftman and al. recently showed an association between multiple risk factors and the occurrence of perioperative cardiovascular complications in renal transplant recipient[27]. Considering the high frequency of cardiovascular disease in our population, we suggest that non-invasive testing be considered in patients presenting two or more risk factors. MPI was performed every 3 years in our population because of good cost-effectiveness, availability and negative predictive value. Prophylactic coronary revascularization is not recommended[9]. More studies are needed to assess the effectiveness of such an invasive approach in renal transplant recipients.
